# Combining transoral fundoplication and robotic cruroplasty: a novel robotic-assisted endoscopy

**DOI:** 10.1055/a-2387-3881

**Published:** 2024-09-04

**Authors:** Maryam Alkhatry, Abdulwahid Alwahedi, Hayder Hammadi

**Affiliations:** 1 Department of Gastroenterology and Endoscopy, Emirates Health Services, Ras Al-Khaimah, United Arab Emirates; 2 Gastroenterology Department, Ibrahim Bin Hamad Obaidullah Hospital, Ras Al Khaimah, United Arab Emirates; 3 222611 General Surgery Department, Al Qassimi Hospital, Sharjah, United Arab Emirates

A 34-year-old man with severe gastroesophageal reflux disease (GERD) and grade D esophagitis combined with a large hiatal hernia did not fully respond to medication and lifestyle changes. As a result, it was decided to pursue a combined endoscopic and surgical approach. This report evaluates the feasibility and effectiveness of robotic-assisted hiatal hernia repair combined with endoscopic transoral incisionless fundoplication (Robo cTIF) 1 2 3 , which combines endoscopy and robotics to treat chronic GERD associated with large hiatal hernias ( Fig. 1 ).

**Fig. 1:**
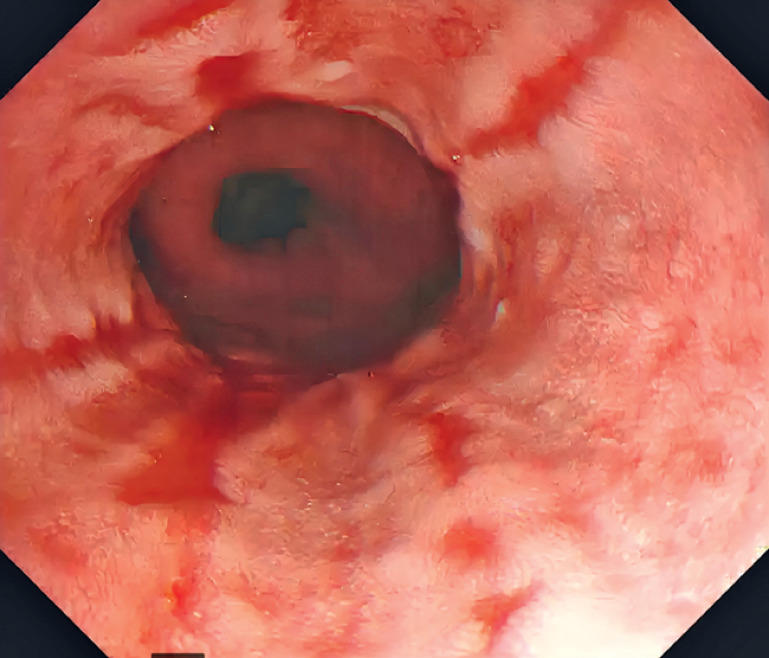
Endoscopy showing hiatal hernia with grade D esophagitis.

In this procedure, the patient underwent robotic-assisted laparoscopic hernia repair followed by endoscopic transoral incisionless fundoplication during a single anesthetic session ( Video 1 ), complemented by a multifaceted intervention that included nutritional counseling and physical activity guidelines. Proton pump inhibitors (PPIs) were tapered off over 6 weeks. We conducted baseline and 3-month assessments to measure endoscopic changes, GERD Health-Related Quality of Life (GERD HRQL) 2 , Reflux Symptom Index (RSI), and post-procedure gastrografin (Bracco Diagnostics Inc., Monroe Township, New Jersey, USA) imaging ( Fig. 2 ).

Combining transoral fundoplication and robotic cruroplasty: a novel robotic-assisted endoscopy.Video 1

**Fig. 2:**
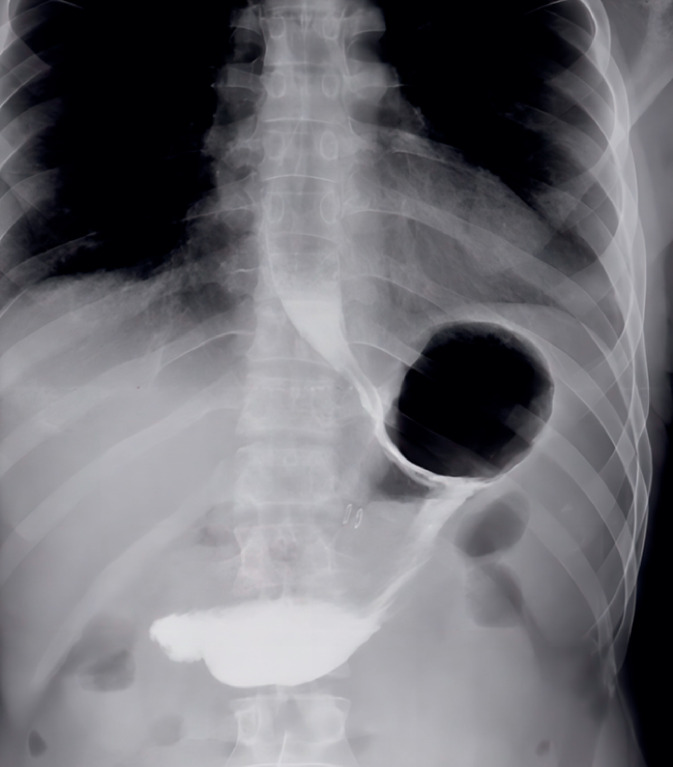
Gastrografin study during swallow 3 months after the procedure.

The procedure commenced with a robotic-assisted hiatal hernia repair cruraplasty capitalizing on the robot’s precision, dexterity, and 3-dimensional visualization ( Fig. 3 ). This was followed by an endoscopic fundoplication using the TIF 2.0 device 4 , to re-establish the gastroesophageal valve ( Fig. 4 ). The procedure was then supported by indocyanine green 5 , ensuring tissue viability post-procedure ( Fig. 5 ).

**Fig. 3:**
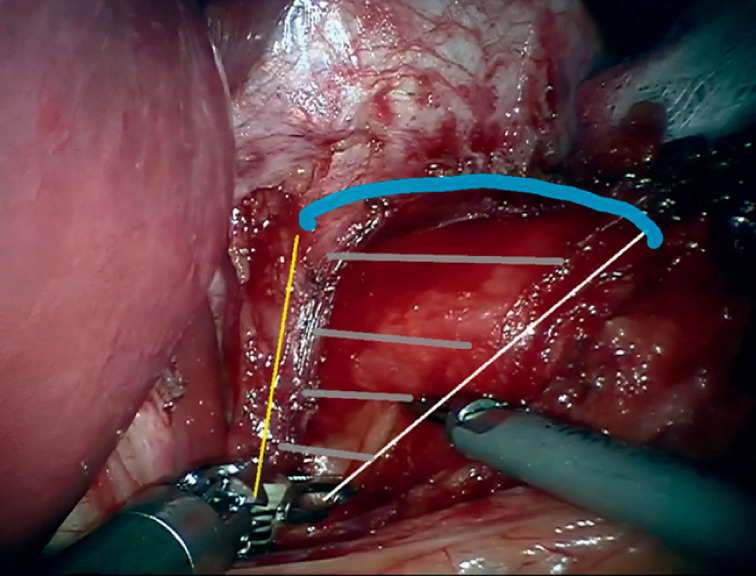
Robotic surgery, hiatal dissection, and mobilizing the esophagus. The upper edge of the hiatal hernia (blue line), and right and left edges of the diaphragm (yellow and white lines) are shown, with the hiatal defect between (sectioned portion).

**Fig. 4:**
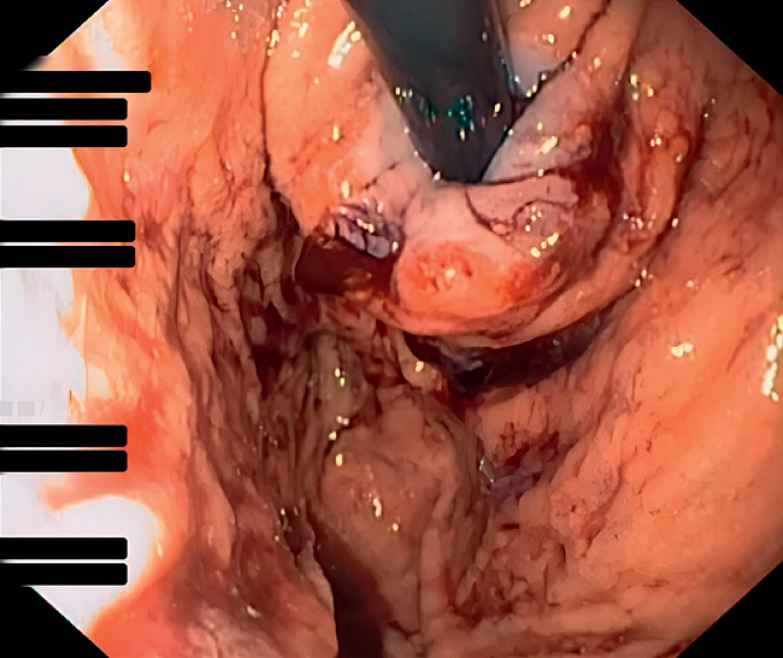
The gastroesophageal valve after transoral incisionless fundoplication.

**Fig. 5:**
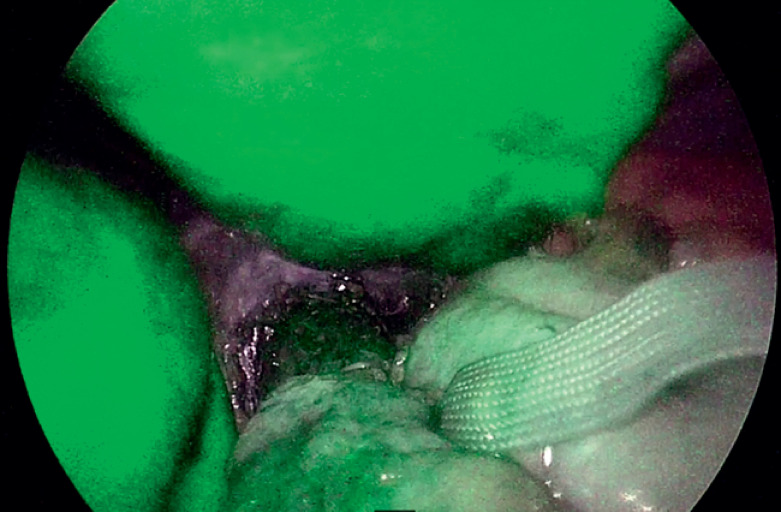
The ribbon marks the gastroesophageal junction. Indocyanine green was used to detect flap-valve vascularity.

Our procedure resulted in significant improvements in all evaluated outcomes from baseline to 3 months post-procedure. GERD HRQL scores greatly improved, dropping from 72 to 1 at 3 months post-procedure. Additionally, the RSI significantly declined from 24.0 to 2 at 3 months, indicating effective management of chronic GERD with hiatus hernia both objectively and subjectively. The patient experienced minimal postoperative pain, rapid recovery, and significant symptom improvement, which eliminated the need for PPIs.

In conclusion, the combined approach of robotic cruroplasty and TIF (Robo cTIF) successfully treated our 34-year-old patient. This method led to significant improvements in quality of life and reflux symptoms, with greatly reduced GERD-HRQL and RSI scores. The patient experienced minimal postoperative pain, rapid recovery, and no longer requires PPIs. This highlights the potential of this technique for severe GERD cases with large hiatal hernias. However, the availability of this innovative approach may be limited to specific candidates who meet certain criteria and largely depends on receiving approval from local health systems.

Endoscopy_UCTN_Code_TTT_1AO_2AJ

## Notes

Endoscopy E-Videos https://eref.thieme.de/e-videos
*E-Videos* is an open access online section of the journal *Endoscopy* , reporting on interesting cases and new techniques in gastroenterological endoscopy. All papers include a high-quality video and are published with a Creative Commons CC-BY license. Endoscopy E-Videos qualify for HINARI discounts and waivers and eligibility is automatically checked during the submission process. We grant 100% waivers to articles whose corresponding authors are based in Group A countries and 50% waivers to those who are based in Group B countries as classified by Research4Life (see: https://www.research4life.org/access/eligibility/ ). This section has its own submission website at https://mc.manuscriptcentral.com/e-videos .

## References

[1] Glenn Michael Ihde II Pena C Scitern C pH scores in hiatal repair with transoral incisionless fundoplication JSLS 2019 23 e2018.00087 10.4293/JSLS.2018.00087 PMC6333564 30675094

[2] Hopkins RJ Irvine T Jamieson GG Long-term follow-up of two randomized trials comparing laparoscopic Nissen 360° with anterior 90° partial fundoplication Br J Surg 2020 107 56 63 31502659 10.1002/bjs.11327

[3] Choi AY Roccato MK Samarasena JB Novel interdisciplinary approach to GERD: concomitant laparoscopic hiatal hernia repair with transoral incisionless fundoplication J Am Coll Surg 2021 232 309 318 33346082 10.1016/j.jamcollsurg.2020.11.021

[4] Janu P Shughoury AB Venkat K Laparoscopic hiatal hernia repair followed by transoral incisionless fundoplication with EsophyX device (HH+ TIF): efficacy and safety in two community hospitals Surg Innovat 2019 26 675 686 10.1177/1553350619869449 PMC6843624 31431138

[5] Paraboschi I Privitera L Loukogeorgakis S Fluorescence-guided surgery (FGS) during a laparoscopic redo Nissen fundoplication: the first case in children Children (Basel) 2022 9 947 35883931 10.3390/children9070947 PMC9325017

